# Supplementary figures and images for: Crystal structure and solution state of the C-terminal head region of the narmovirus receptor binding protein
Source: mBio. 2023 Sep 22;14(5):e01391-23. doi: 10.1128/mbio.01391-23 (PMC10653815; doi:10.1128/mbio.01391-23)

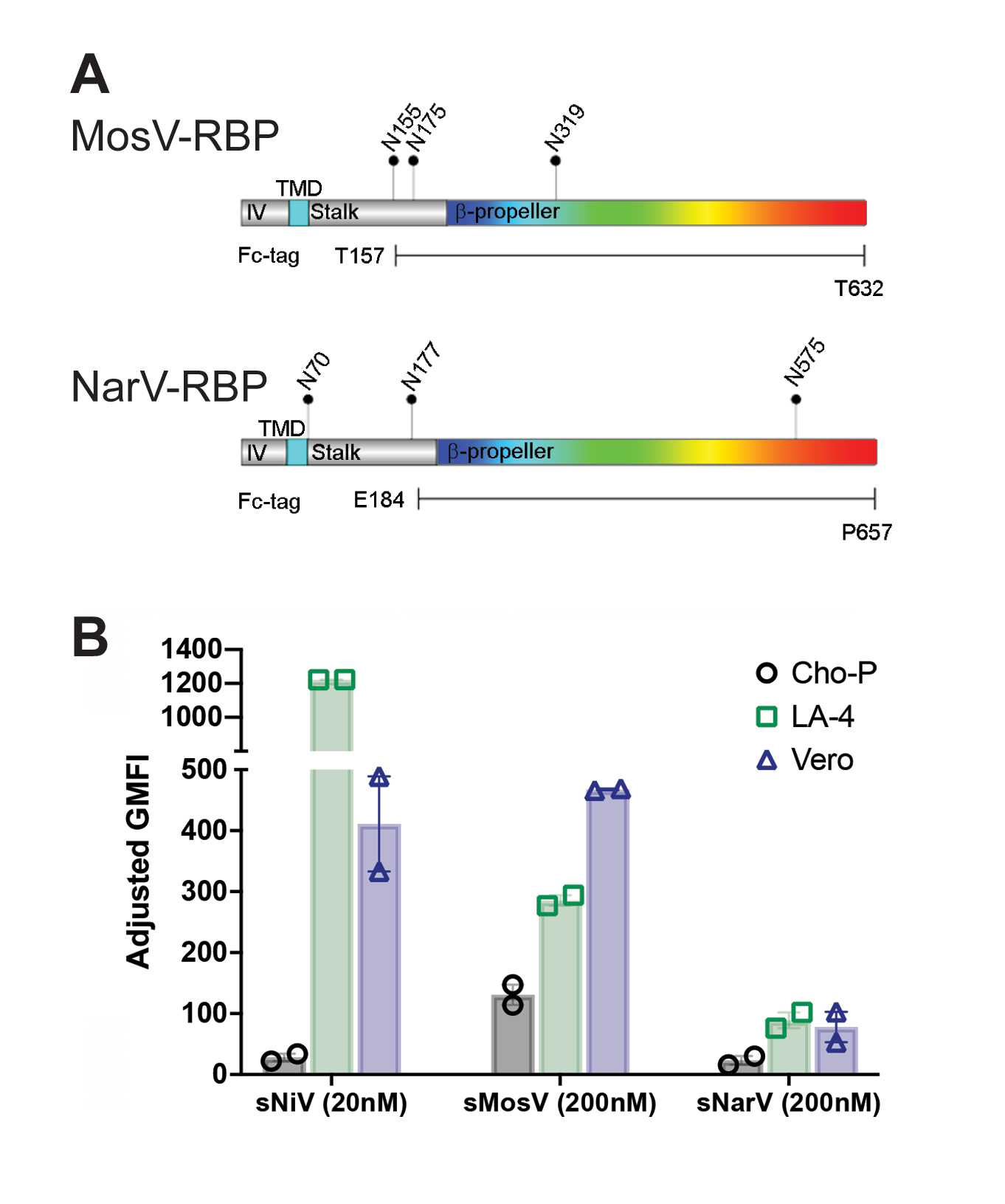

Supplement: Fig. S1 — Binding analysis of soluble MosV-RBP and NarV-RBP to a panel of cell-types. (A) Gene diagram generated with DOG2.0 displaying features of the MosV and NarV RBPs, including the intraviral (IV, gray) region, transmembrane domain (TMD, cyan), stalk region (gray), and six-bladed β-propeller receptor binding head (rainbow). Predicted N-linked glycosylation sites (NXS/T, where X≠P) are marked with pins and relevant asparagine residue numbered. The lengths of the Fc-tagged MosV-RBP and NarV-RBP constructs used in the cell binding analysis are shown below the gene diagram. (B) Soluble receptor binding protein (RBP) binding to LA-4, Vero, and CHO pgsA745 cell lines. The indicated concentration of soluble, Fc-tagged RBP was incubated with cells, then stained with APC-tagged anti-Fc secondary antibody and subjected to flow cytometry as described in the methods. Adjusted geometric mean fluorescence intensity (GMFI) was calculated as a product of the percent APC positive cells and the GMFI of the APC positive cells. Shown are the results of two replicates for each cell line with error bars representing the standard error of the mean. MosV-RBPβ showed moderate binding and NarV-RBPβ showed low level binding to both LA-4 and Vero cells. Additionally, MosV-RBPβ showed a low level of binding to the CHO pgsA745 cells. The decreased binding affinity of NarV-RBPβ could be attributed to the shorter construct length. [file mbio.01391-23-s0001.tif]

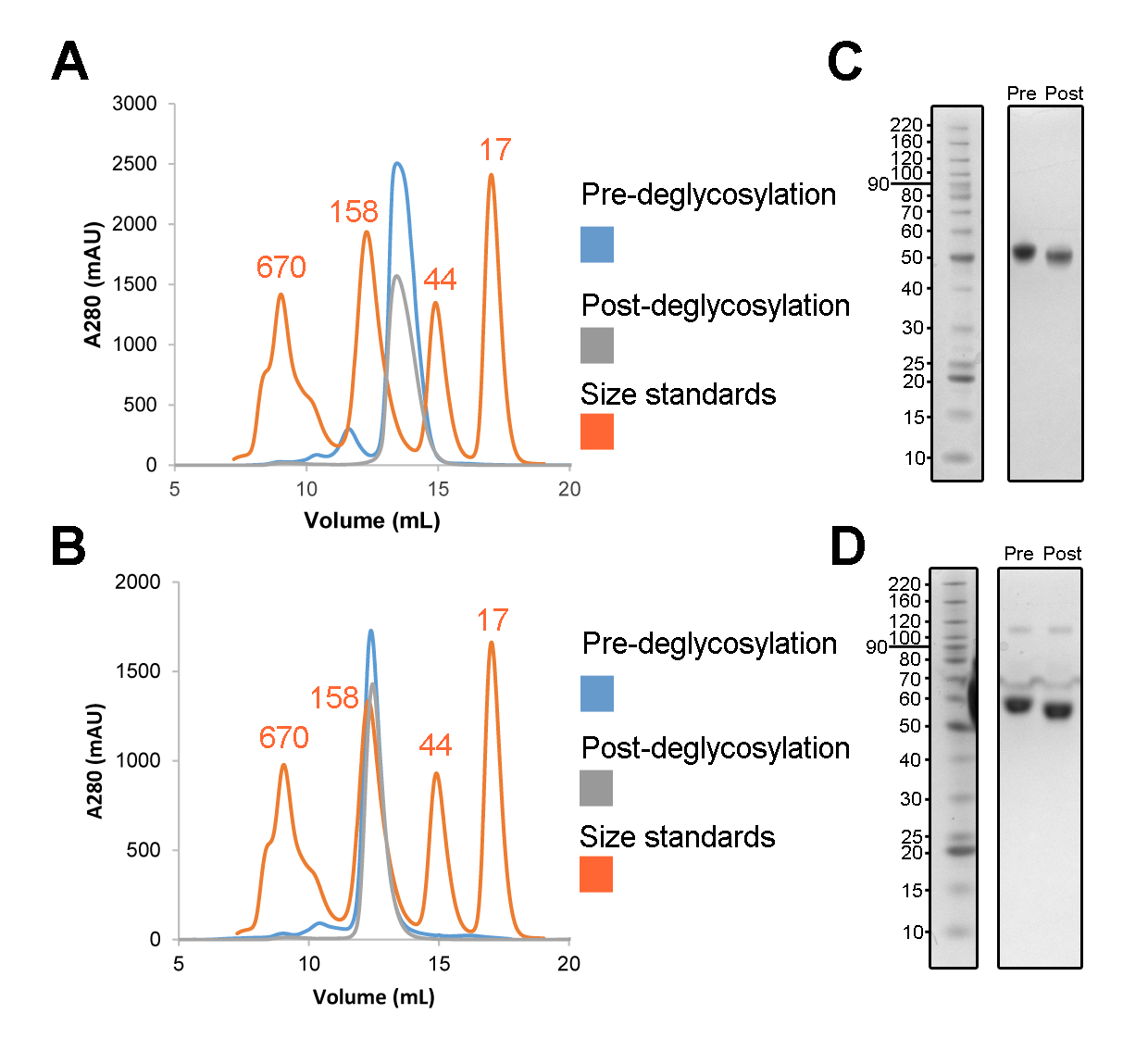

Supplement: Fig. S2 — Size exclusion chromatograms of MosV-RBPβ and NarV-RBPβ. (A) MosV-RBPβ and (B) NarV-RBPβ expressed transiently in the presence of kifunensine, pre- (blue) and post-deglycosylation (grey). Samples were run across a Superdex™ 200 10/30 column (Cytvia) and compared with gel filtration standards (orange; BioRad). The expected size of the monomeric and dimeric MosV-RBPβ was 49 kDa and 98 kDa, respectively. The size standards eluted from the column in the following order: thyroglobulin (670 kDa), β-globulin (158 kDa), ovalbumin (44 kDa), myoglobulin (17 kDa), vitamin B12 (1.35 kDa). The calculated molecular weight of monomeric MosV-RBPβ is 49 kDa, the species observed through SEC analysis (44 kDa−158 kDa) would be consistent with a dimeric arrangement of MosV-RBP (98 kDa). The expected molecular weight of NarV-RBP, is 53 kDa, however the species observed with SEC analysis is much larger, displaying a size consistent with the 158 kDa gel filtration size standard. This is larger than the expected size of the monomer, consistent with hypothesis that NarV-RBP forms a stable higher order oligomer in solution (dimer 106 kDa; trimer 159 kDa). Prior to crystallization the protein was treated with EndoF1 to remove high-mannose glycoforms. SDS-PAGE analysis of the purified (C) MosV-RBPβ and (D) NarV-RBPβ pre- and post-deglycosylation, shows a shift following treatment with EndoF1. [file mbio.01391-23-s0002.tif]

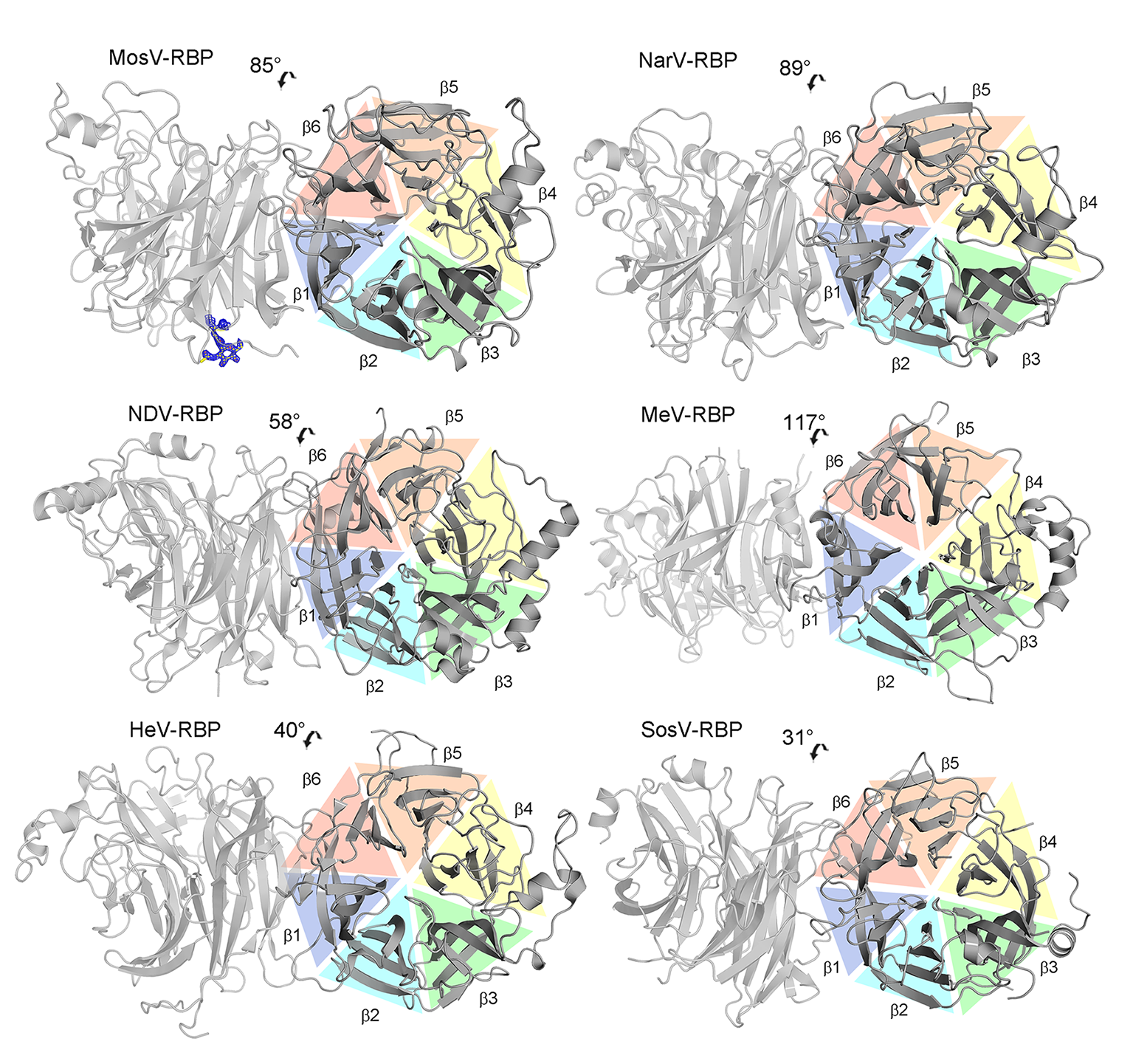

Supplement: Fig. S3 — Representative paramyxoviral RBP homodimeric interfaces. The crystallographic dimers of NDV-RBPβ (1E8V), SosV-RBPβ, HeV-RBPβ (2X9M) and MeV-RBPβ (3INB) are shown in cartoon representation (grey). The angle of association, calculated using UCSF Chimera, is shown above the dimer. [file mbio.01391-23-s0003.tif]

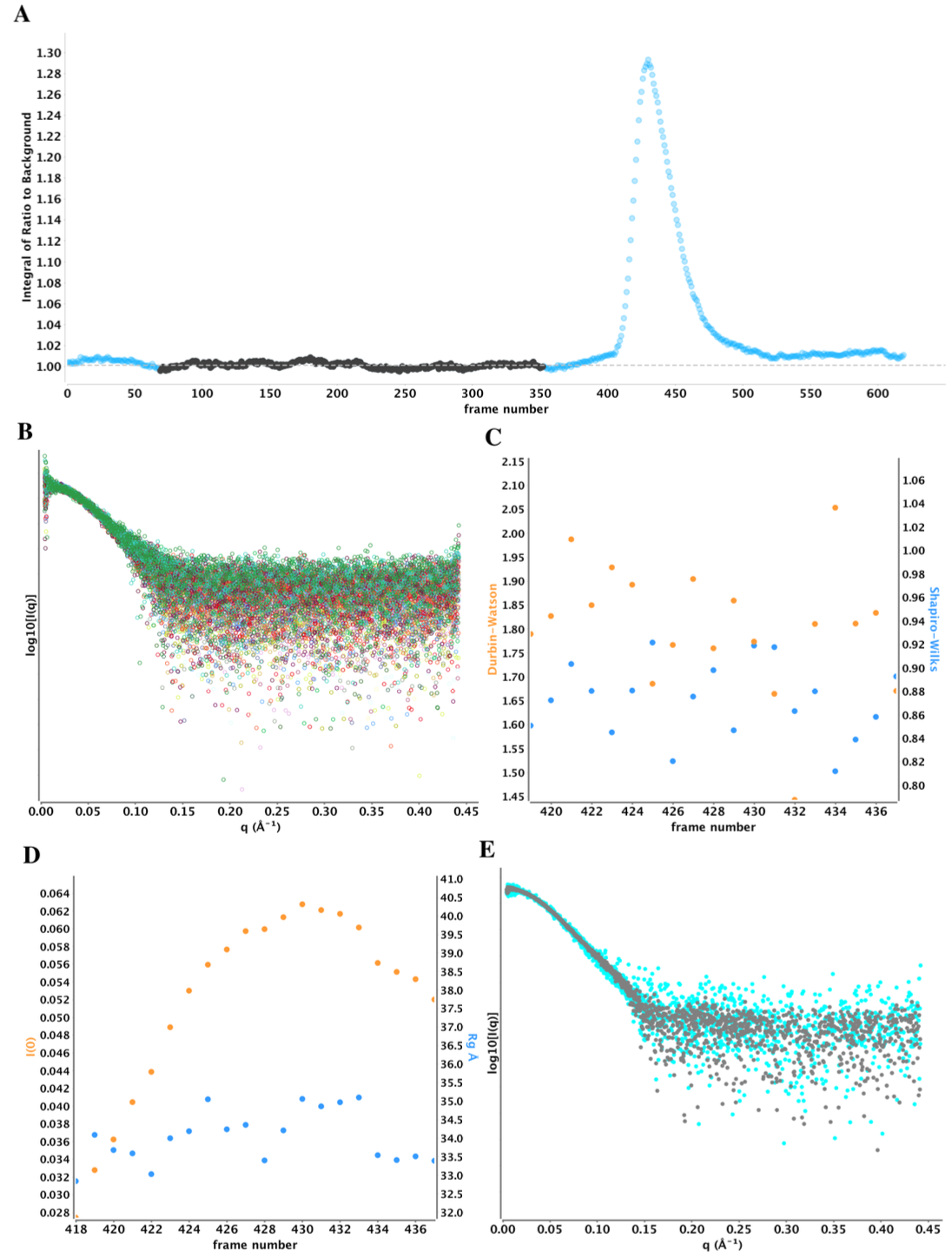

Supplement: Fig. S4 — MosV-RBPβ SEC-SAXS summary. (A) SEC-SAXS Signal Plot. Each point is the integrated area of the ratio of the sample SAXS curve to the estimated background (gray). Averaged background horizontal dashed line. (B) Overlay of subtracted SAXS curves used in final averaged curve (frames 418 to 437). Each frame is scaled to peak. (C) Durbin-Watson and Shapiro-Wilks tests examining the distribution of the residuals between two frames. In this case, comparisons are made in reference to the first frame. Radiation damage or lack of similarity can be observed as a trend in either statistic across the frame set. Likewise, similarity is demonstrated by a random distribution of the statistics. (D) Double Y plot with I(0), orange, and Rg, cyan, estimated from the Guinier region for each subtracted frame. For a single concentration measurement made over several frames, radiation damage will be observed as an increase in I(0) and Rg. For SEC-SAXS, I(0) should change with the concentration of the particle during elution. (E) Log 10 intensity plot of subtracted and merged SAXS frames. Black represents averaged buffer frames subtracted from averaged sampled frames. Cyan represents median of the buffer frames subtracted from the averaged sample frames. Poor buffer subtraction leads to a displacement between the two curves at high-q. [file mbio.01391-23-s0004.tif]

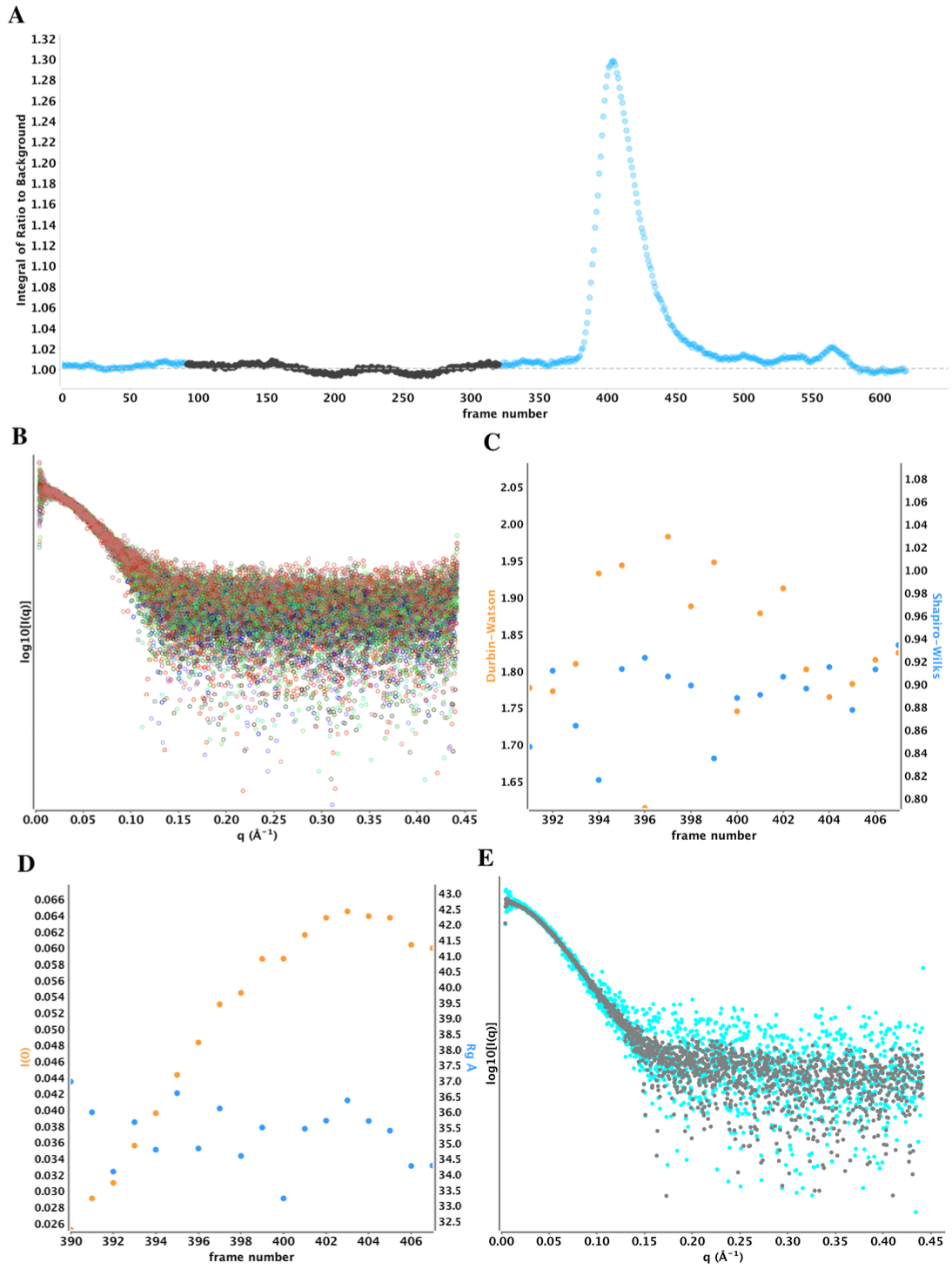

Supplement: Fig. S5 — NarV-RBPβ SEC-SAXS summary. (A) SEC-SAXS Signal Plot. Each point is the integrated area of the ratio of the sample SAXS curve to the estimated background (gray). Averaged background horizontal dashed line. (B) Overlay of subtracted SAXS curves used in final averaged curve (frames 418 to 437). Each frame is scaled to peak. (C) Durbin-Watson and Shapiro-Wilks tests examining the distribution of the residuals between two frames. In this case, comparisons are made in reference to the first frame. Radiation damage or lack of similarity can be observed as a trend in either statistic across the frame set. Likewise, similarity is demonstrated by a random distribution of the statistics. (D) Double Y plot with I(0), orange, and Rg, cyan, estimated from the Guinier region for each subtracted frame. For a single concentration measurement made over several frames, radiation damage will be observed as an increase in I(0) and Rg. For SEC-SAXS, I(0) should change with the concentration of the particle during elution. (E) Log 10 intensity plot of subtracted and merged SAXS frames. Black represents averaged buffer frames subtracted from averaged sampled frames. Cyan represents median of the buffer frames subtracted from the averaged sample frames. Poor buffer subtraction leads to a displacement between the two curves at high-q. [file mbio.01391-23-s0005.tif]

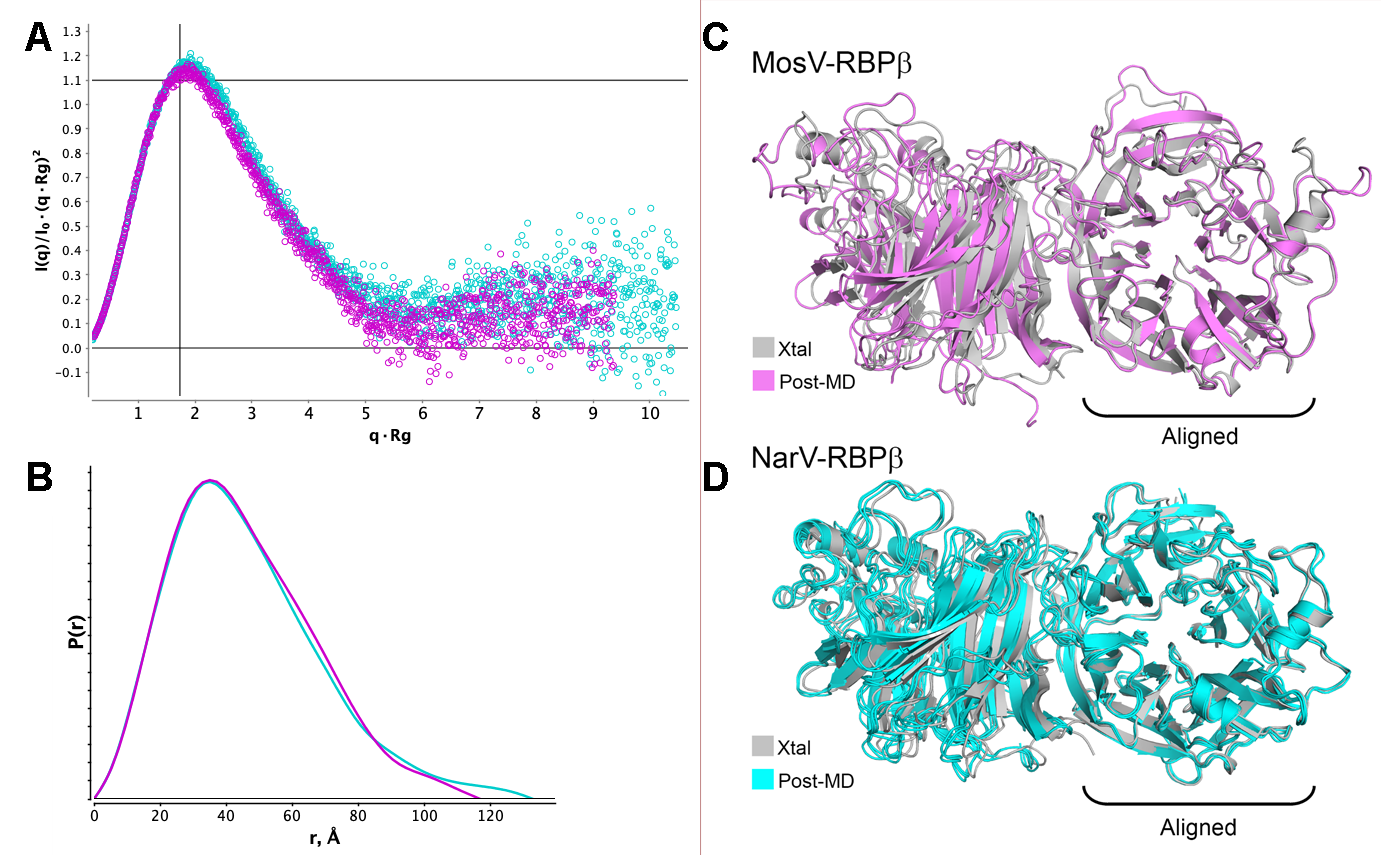

Supplement: Fig. S6 — SAXS Summary plots for MosV-RBPβ and NarV-RBPβ and alignment of narmovirus β-propeller structures pre- and post-molecular dynamics (MD). (A) Dimensionless Kratky Plot. Crosshairs denote Guinier-Kratky plot (peak position for an ideal globular particle). Convergence to baseline and peak position supports MosV (violet) and NarV (cyan) RBP β-propellers are compact but not ideally globular particles. (B) Pair-distance distribution function from indirect Fourier transform of datasets described in table S3). Plots were prepared using the program ScÅtter (www.bioisis.net). MosV-RBPβ (C) and NarV-RBPβ (D) post-MD dimers were aligned to a single protomer of the crystallographic dimer. For this analysis only the b-propellers were aligned, with the GlcNAc2Man9 glycans and additional residues at the termini being omitted. The MosV-RBPβ and NarV-RBPβ structures are shown in cartoon representation, with the crystallographic dimers coloured gray, and the post-MD dimers coloured violet and cyan, respectively. [file mbio.01391-23-s0006.tif]

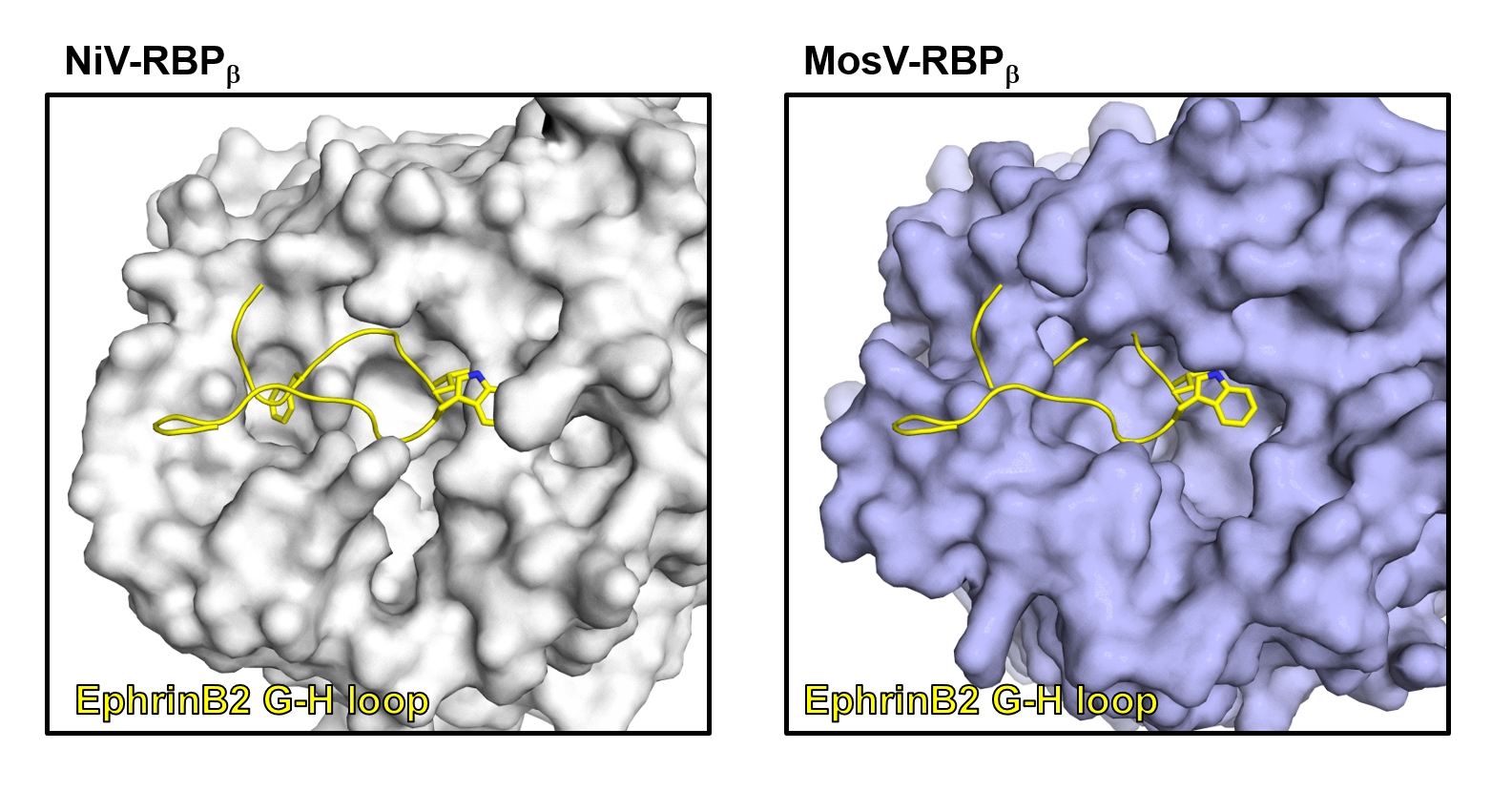

Supplement: Fig. S7 — The surface of MosV-RBP is likely unable to accommodate the G-H loop of B-type ephrin ligands. The surface of NiV-RBP (left) (white), with the G-H loop of ephrinB2 shown in cartoon representation (yellow) (2VSM) [2]. To the right MosV-RBP (blue) is shown in surface representation with the G-H loop docked into the putative binding site, revealing that the loop would be unlikely to bind due to the lack of the necessary pocket. [file mbio.01391-23-s0007.tif]
