# Supplementary material for: Crystal structure and solution state of the C-terminal head region of the narmovirus receptor binding protein
Source: mBio. 2023 Sep 22;14(5):e01391-23. doi: 10.1128/mbio.01391-23 (PMC10653815; doi:10.1128/mbio.01391-23)
Supplement: Supplemental tables — Tables S1 to S3 [file mbio.01391-23-s0008.pdf]

Table S1: Data collection and refinement statistics for MosV-RBP$_{\beta}$.

	Native	Pt Peak	High resolution native
**Data collection statistics**			
**Beamline**	DLS I02	DLS I04	DLS I04
**Spacegroup**	$I\ 4_1\ 2\ 2$	$I\ 4_1\ 2\ 2$	$P\ 2_1\ 2_1\ 2_1$
**Cell dimensions**			
**a, b, c (Å)**	214.0, 214.0 138.4	214.0, 214.0 136.2	80.2, 83.9, 122.1
**α, β, γ (°)**	90.0, 90.0, 90.0	90.0, 90.0, 90.0	90.0, 90.0, 90.0
**Resolution range (Å)**	67.66–2.75 (2.82–2.75)[a]	63.22–3.24 (3.32–3.24)	69.17–1.62 (1.65–1.62)
**Wavelength (Å)**	0.9795	1.0721	0.9795
**R_{merge}**	0.12 (2.26)	0.18 (4.63)	0.08 (1.47)
**R_{meas}**	0.13 (2.46)	0.20 (4.76)	0.08 (1.62)
**R_{pim}**	0.04 (0.79)	0.03 (0.65)	0.03 (0.68)
**I/σ I**	15.3 (1.3)	22.1 (1.2)	13.0 (1.1)
**$CC_{1/2}$**	1.00 (0.51)	1.00 (0.56)	1.00 (0.50)
**Completeness (%)**	99.7 (99.7)	99.9 (100.0)	99.8 (99.4)
**Multiplicity**	11.7 (12.0)	53.6 (53.8)	6.5 (5.5)
**Wilson B factor (Å^2)**	82	115	24
**Total observations**	487,544 (36,528)	1,359,652 (99,567)	678,469 (28,239)
**Total unique**	41,704 (3,054)	25,387 (1,851)	105,014 (5,168)

**Refinement statistics**	
**Resolution (Å)**	48.58–1.62 (1.68–1.62)
**R_{work} / R_{free}[b] (%)**	17.3/20.1
**Rfree test set**	5029 (4.8%)
**No. atoms**	
**Protein**	6,766
**Ligand/ion**	14
**Water**	695
***B*–factors**	
**Protein**	33
**Ligand/ion**	36
**Water**	39
**R.m.s deviations[c]**	
**Bond lengths (Å)**	0.006
**Bond angles (°)**	0.89
**Ramachandram analysis[d]**	
**Favored region (%)**	96.3
**Allowed region**	3.4

[a]Numbers in parentheses refer to the outer resolution shell.
[b]R_{free} is calculated as for R_{work}, but using only 5% of the data which were separated prior to refinement.
[c]r.m.s deviations: root mean square deviation from ideal geometry.
[d]Determined using the Molprobity server (68).

Table S2: Data collection and refinement statistics for NarV-RBP$_\beta$.

**Data collection statistics**	
**Beamline**	DLS I04
**Spacegroup**	P 1 2_1 1
**Cell dimensions**	
**a, b, c (Å)**	55.4, 82.9, 93.1
**α, β, γ (°)**	90.0, 90.9, 90.0
**Resolution range (Å)**	82.96–2.07 (2.14–2.07)[a]
**Wavelength (Å)**	0.9795
**R_{merge}**	0.18 (0.81)
**R_{meas}**	0.24 (1.09)
**R_{pim}**	0.13 (0.59)
**I/σ I**	4.2 (1.3)
**$CC_{1/2}$**	0.98 (0.62)
**Completeness (%)**	100 (99.9)
**Multiplicity**	3.3 (3.4)
**Wilson B factor (Å^2)**	18
**Total observations**	169,452 (12,479)
**Total unique**	49,851 (3,683)
**Refinement statistics**	
**Resolution (Å)**	55.38–2.07 (2.14–2.07)
**R_{work} / R_{free}[b] (%)**	23.0/27.0
**Rfree test set**	2607 (5.1%)
**No. atoms**	
**Protein**	6,702
**Ligand/ion**	0
**Water**	721
***B*-factors**	
**Protein**	23
**Ligand/ion**	N/A
**Water**	27
**R.m.s deviations[c]**	
**Bond lengths (Å)**	0.003
**Bond angles (°)**	0.63
**Ramachandran analysis[d]**	
**Favored region (%)**	97.1

[a]Numbers in parentheses refer to the outer resolution shell.
[b]R_{free} is calculated as for R_{work}, but using only 5% of the data which were separated prior to refinement.
[c]r.m.s deviations: root mean square deviation from ideal geometry.
[d]Determined using the Molprobity server (68).

Table S3. SAXS data collection statistics.

	MosV-RBP$_\beta$	NarV-RBP$_\beta$
**Data collection statistics**		
q_{min} (Å^{-1})	0.00641589	0.01293878
q_{max} (Å^{-1})	0.286417	0.310575
$R_{g,\ reciprocal}$ (Å^{-1})	34.4 (±0.49)	34.75 (±0.42)
$R_{g,real}$ (Å^{-1})	35.20	36.10
Porod Volume (Å^3)	179,441	181,994
V_c (Å^2)	655.3	623.0
Porod Exponent	3.99 (±0.20)	3.99 (±0.21)
d_{max} (Å)	117	133
N_s	11	14
Number of points*	1130	1234
SEC-column	Shodex KW-403	
Flow rate	160 uL per minute	
Temperature (K)	298	

* Represents total number of points used in real-space transform and in the fitting to the atomistic models. D_{max} refers to maximum dimension of the particle determined using the real-space indirect transform in ScÅtter (www.bioisis.net).
